# Spatiotemporal Pattern of Acid Phosphatase Activity in Soils Cultivated With Maize Sensing to Phosphorus-Rich Patches

**DOI:** 10.3389/fpls.2021.650436

**Published:** 2021-04-13

**Authors:** Xiaofan Ma, Haigang Li, Junling Zhang, Jianbo Shen

**Affiliations:** ^1^College of Resources and Environmental Sciences, China Agricultural University, Beijing, China; ^2^Inner Mongolia Key Laboratory of Soil Quality and Nutrient Resources, Key Laboratory of Grassland Resource (IMAU), Ministry of Education, College of Grassland, Resources and Environment, Inner Mongolia Agricultural University, Hohhot, China

**Keywords:** acid phosphatase, diurnal rhythm, maize, P-rich patches, soil zymography

## Abstract

**Aims:**

Acid phosphatase (APase) secretion by roots allows plants to mobilize organic phosphorus (P) in low P soils. However, the spatiotemporal dynamics of soil APase activity in response to P-rich patches remain unclear.

**Methods:**

Here, we grew maize in rhizoboxes with two contrasting soil types and different localized P supplies. *In situ* soil zymography was applied to examine the spatial-temporal variation of APase activity.

**Results:**

We found P-rich patches can induce the secretion of APase from roots, indicating that even mineral P fertilizers were localized apply, mobilization of soil organic P by roots can also be enhanced; APase hotspot areas and APase activities in the rhizosphere and bulk soil of the same rhizobox showed opposite diurnal rhythms across the whole soil profile. The APase hotspot area was 10–140% larger at noon than at midnight in the rhizosphere, which is consistent with the diurnal rhythm of photosynthesis. In contrast, in bulk soil, the area was 18–200% larger at midnight than at noon, which led to spatiotemporal niche differentiation with regard to the utilization of soil organic P; this alleviated competition between plants and soil microorganisms.

**Conclusion:**

Our findings showed that APase secretion of roots was plastic in P-rich patches and showed an opposite diurnal rhythm with soil microorganisms in bulk soil.

## Introduction

Phosphorus (P) is a vital macronutrient for plant growth and development (Tiessen, 2008; Cordell et al., 2009). It is not only a component of organic compounds, including nucleic acids and ATP, in plants but also participates in the metabolism of various substances and energy, regulates physiological and biochemical processes, and plays an irreplaceable role in plant growth and yield (Stryker et al., 1974; Schachtman et al., 1998; Vance et al., 2003). However, owing to the low diffusion coefficient of soil P, which is inclined to be immobilized by minerals containing Ca^2+^, Fe^3+^, and Al^3+^, the bioavailability of soil P is very low in most soils (Hinsinger, 2001). P deficiency has reduced crop yields by approximately 30% around the world (Vance et al., 2003), and in China, at least half of the arable soil Olsen-P levels are below the level for optimal crop production (Zhang et al., 2019).

Organic P, as an important soil P source for plants, constitutes 20–80% of soil total P (Anderson, 1980) and it cannot be directly absorbed by roots unless hydrolyzed by phosphatase into phosphate anions. It was well-documented that P deficient plants enhance acid phosphatase (APase) secretion from roots into rhizosphere to hydrolyze organic P, which is an important adaptation strategy for plants grown in low P soils (Yadav and Tarafdar, 2001; George et al., 2002; Tomscha et al., 2004). Microorganisms, in addition to roots, are another important source of APase in rhizosphere (Marschner, 2013; Wei et al., 2018). Many factors can influence APase activity and the diffusion range in rhizosphere, such as soil moisture, temperature, plant nutrition status and fertilizers application (Poll et al., 2006; Kuzyakov and Blagodatskaya, 2015; Xu et al., 2017). Although APase cannot mobilize soil inorganic P, it was still induced by mineral P fertilizers application when plants are suffering P deficient stress (Khan, 1970; Spiers and Mcgill, 1979). Soil P is heterogenetic distribution along soil profiles, where many P-rich patches form due to mineral P fertilizers application. Up to now, few studies have focused on the response of APase activity to heterogenetic distribution of soil P because of methodology shortage.

Soil zymography, a recently developed non-destructive *in situ* technology for two-dimensional imaging, has been successfully implemented for the determination of various enzyme spatial distributions along roots and across rhizosphere-bulk soil gradients (Giles et al., 2018; Guber et al., 2018; Wei et al., 2019). Apart from spatial variation, many rhizosphere processes, such as phytosiderophore secretion, rhizosphere acidification and carboxylate exudation, also show temporal variability (Walter et al., 1995; Neumann et al., 1999; Rudolph et al., 2013). Blossfeld et al. (2013) quantified the diurnal pattern of rhizosphere pH with planar optodes. Soil zymography makes it possible to determine the diurnal pattern of APase activity in soil.

Maize is one of the most important crops around the world, providing biofuel, forage, and human and animal food (Wen et al., 2017; Azeem et al., 2018). Many previous studies have shown that P-deficient maize can modify its morphological and physiological processes to increase P uptake from soil (Kummerova, 1986; Jakobsen et al., 2005; Corrales et al., 2007; Zhang et al., 2012). However, spatiotemporal changes in soils cultivated with maize sensing to P-rich patches along soil profile are still lacking. Here, we hypothesized that (1) APase activity in the rhizosphere is enhanced by P-rich patches, and (2) the activity shows a diurnal pattern. We tested the above two hypotheses in typical maize-cultivated soils in China, including calcareous soil and acid soil.

## Materials and Methods

### Plants, Soil, and Experimental Design

The fluvo-aquic soil (calcareous soil) sample was taken from the top 20 cm of the profile in a field at the Changping Experimental Station of China Agricultural University, Beijing (40°01′N, 116°17′E). The initial soil properties were as follows: pH 8.40 (1:2.5, soil:water), organic carbon 11.5 g kg^–1^, total N 0.72 g kg^–1^, N_min_ 0.51 g kg^–1^, Olsen-P 1.68 mg kg^–1^, NH_4_OAc-K 82.4 mg kg^–1^. The red soil (acidic soil) was collected from the top 20 cm of the cultivation layer at the Boluo experimental station, South China Agricultural University, Huizhou, Guangdong (23°18′N, 114°28′E). The properties of the initial soil were as follows: pH 4.7 (1:2.5, soil:water), organic carbon 14.6 g kg^–1^, total N 0.37 g kg^–1^, Olsen-P 2.3 mg kg^–1^, NH_4_OAc-K 27.4 mg kg^–1^. All the samples were air-dried and thoroughly mixed after removal of plant residues and stones and then sieved to 2 mm. To ensure an adequate supply of other nutrients for maize (*Zea mays* L. cv. ZD 958) growth, soil was supplemented with basic nutrients at the following rates (mg kg^–1^ soil): CO(NH_2_)_2_ 200, K_2_SO_4_ 100, CaCl_2_⋅2H_2_O 200, MgSO_4_⋅7H_2_O 50, EDTA-FeNa 5, MnSO_4_⋅4H_2_O 5, ZnSO_4_⋅7H_2_O 5, CuSO_4_⋅5H_2_O 5, H_3_BO_3_ 0.68, Na_2_MoO_4_⋅5H_2_O 0.12. Phosphorus was added to the soil at a rate of 200 mg P kg^–1^ soil as calcium superphosphate (SSP, Sinopharm Chemical Reagent Co., Ltd.) and diammonium hydrogen phosphate (DAP, Sinopharm Chemical Reagent Co., Ltd.). Five P treatments were set as follows: (1) no P addition (control); (2) homogenized SSP application to the whole soil (SSP Hom); (3) localized SSP application to patches at the same rate (SSP Pat); (4) homogenized DAP application to the whole soil (DAP Hom); and (5) localized DAP application to patches at the same rate (DAP Pat). P-rich patches were circles with a diameter of 4 cm that were located 4 cm from the top and right side of the rhizobox (20 cm × 1.5 cm × 30 cm) (Supplementary Figure 1).

Each rhizobox had a removable side and contained 1.4 kg air-dried soil. The P-rich patches were placed at the planned locations before the removable side was closed. After 24 h of germination on moist filter papers, one vigorous seed was planted into the center of the rhizobox at a depth of 6 mm. There were four replicates for each treatment. The experiment was carried out in a natural-light glasshouse with a controlled temperature of 20–25°C during the day and 15–18°C at night in China Agricultural University, Beijing (40°01′N, 116°17′E). During maize growth, rhizoboxes were irrigated to 75% field capacity by weight and tilted 45° to ensure that roots grew along the removable sides.

### Sampling and Analysis of Biomass, Root Length, and P Concentration

Plants were harvested at 30 days after sowing. At harvest, the shoots were cut off just above the soil surface, dried at 105°C for 30 min and then dried at 70°C for 48 h, weighed and ground into powder for chemical analysis. All visible roots in each rhizobox were removed and washed with deionized water and then stored at 4°C until root scanning. Roots in all treatments were separated into three parts: (1) P-rich patches for P Pat treatments and the same locations for control and P Hom treatments; (2) contrast areas, a circle (4 cm diameter) located 4 cm from the top and left side of rhizoboxes; and (3) the remaining parts. The washed roots were arranged in a transparent tray and then scanned at 300 dpi resolution (Epson Expression 1600 pro, Model EU-35, Japan). The scanned images were analyzed by a Win-RHIZO image analysis system (Win-RHIZO Pro 2004b, Version 5.0, Canada), and root morphological parameters such as root length and average root diameter were obtained. Roots were also dried at 70°C for 48 h, weighed and ground. The plant materials were digested with H_2_O_2_ and concentrated H_2_SO_4_ for P concentration determination by using the vanadomolybdate method (Westerman, 1990).

### Soil Zymography

After cultivation of the maize for 10 and 20 days, soil zymography, a non-destructive *in situ* technique, was performed as described by Razavi et al. (2016). The basic principle is that polyamide membranes (20 × 20 cm, 0.45 μm; Taoyuan, China) are saturated with 4-methylumbelliferone (MUF) substrates, which fluoresce when enzymatically hydrolyzed (Dong et al., 2007; Wei et al., 2018). 4-Methylumbelliferone-phosphate (MUF-P) was used for visualization of APase activity. MUF-P was dissolved to a concentration of 10 mM in MES buffer (pH 6.7, Sigma-Aldrich, Germany) (Koch et al., 2007), and then the freshly prepared membranes saturated with substrate solution were applied directly to the soil-root surface. After incubation for 1 h in darkroom (Hoang et al., 2016; Liu et al., 2017), membranes were carefully removed from the soil surface, and any attached soil particles were gently cleared using tweezers. After the membranes were placed under UV light with an excitation wavelength of 365 nm (Sankyo, Japan), a photograph was taken by a camera (EOS 5D, Canon).

To quantify the APase activity with photographs, a standard calibration was prepared using membranes (2 cm × 2 cm) soaked in MUF solutions with a concentration gradient (0, 0.01, 0.05, 0.1, 0.5, 1, 3, 6, 8, 10 mol L^–1^). The amount of MUF per unit area was calculated from the solution volume taken by the membranes and its size (Razavi et al., 2016; Ma et al., 2018). The membranes used for the standard curve were photographed under UV light under the same conditions as the samples.

### Image Processing and Analysis

The fluorescence visualized on the zymograms under UV light revealed the areas in which substrates had been degraded by APase. The intensity of fluorescence was linear with enzyme activity. The zymograms were processed and analyzed by the open source software ImageJ. All digital images were first converted to 16-bit grayscale images to correct the noise from the environment and camera. Then, the background (reference object embedded in all images) value and calibration curve at zero MUF concentration were subtracted from all zymograms. Consequently, a linear correlation was determined between APase activity and the gray value on each 4 cm^2^ calibration membrane corresponding to the MUF concentrations. Then, the gray value of each zymography pixel was translated to APase activity (pmol cm^–2^ h^–1^). The root axis was defined as the midpoint between the two observable boundaries (Ge et al., 2019), and the root boundaries were distinguished using the thresholding method in ImageJ. Based on this and previous studies (Ge et al., 2019), we defined the area where the mean APase activity was at least 5% higher than that in the rhizosphere as a rhizosphere hotspot area and the area where the mean enzyme activity was more than 30% higher than that in bulk soil as a bulk soil hotspot area.

Significant differences in acid phosphatase activity, hotspot area, plant biomass, root length, and P concentration between P-treatments were conducted by one-way ANOVA followed by Duncan’s test, and significant differences between noon and midnight were determined by *t*-tests using SPSS statistical software at the *P* ≤ 0.05 probability level.

## Results

### Plant Growth and P Uptake

**FIGURE 1:**
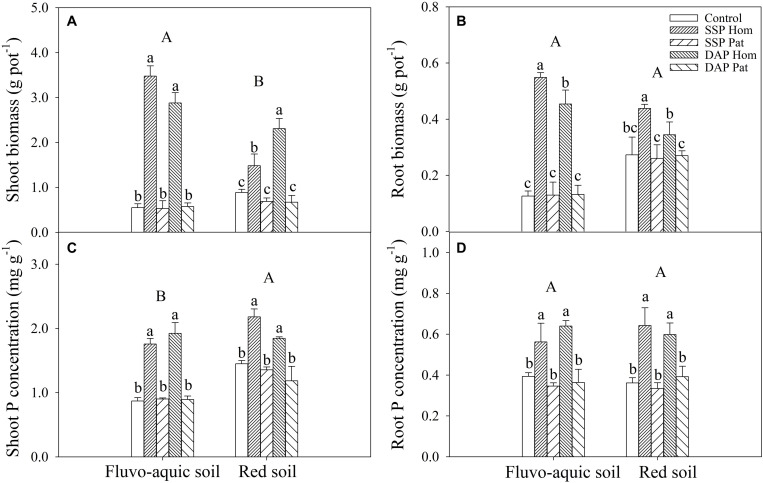
Shoot and root biomass **(A,B)** and P concentration **(C,D)** of maize on 30 DAS in fluvo-aquic soil and red soil. Capital letters indicate significant differences between different soil types, and lower-case letters indicate significant differences between fertilization treatments. Each column refers to the mean value of four replicates (+SD).

**FIGURE 2:**
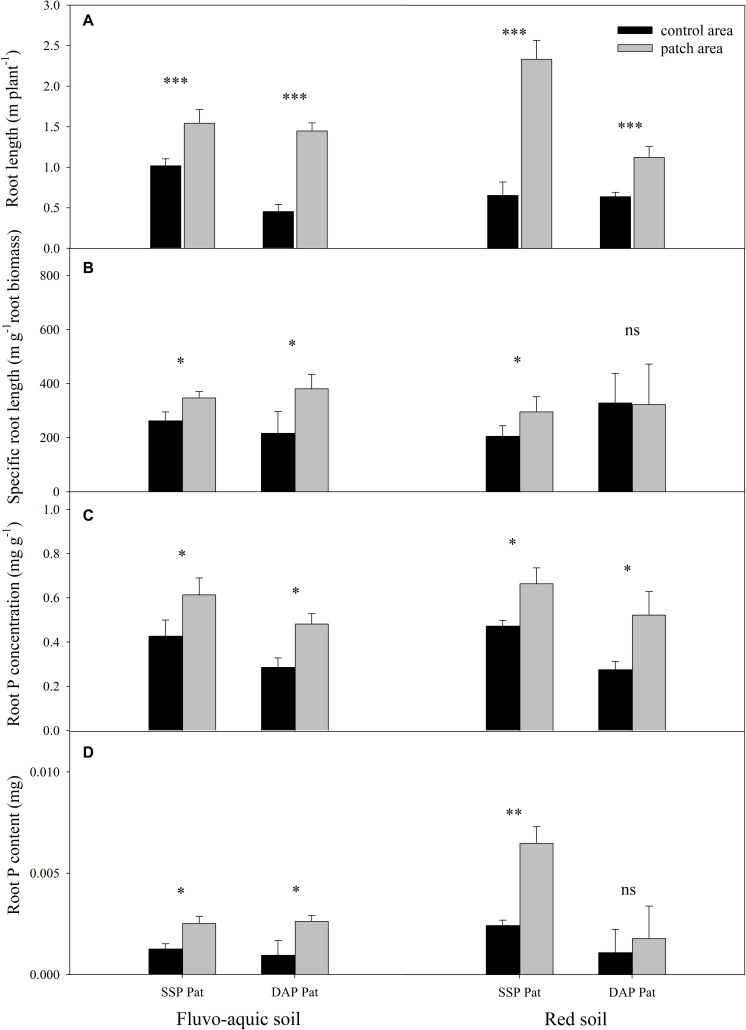
Root length **(A)**, specific root length **(B)**, root P concentration **(C)**, and content **(D)** of maize in P-rich patches and contrast areas on 30 DAS in fluvo-aquic soil and red soil with localized P supply. A set of columns (black and gray) indicates sampled from the same rhizobox. Asterisks indicate significant differences between P-rich patches and contrast areas in the same rhizobox (significance level: ^∗^*P* < 0.05; ^∗∗^*P* < 0.01; ^∗∗∗^*P* < 0.001; ns, no significant). Each column refers to the mean values of four replicates (+SD).

**FIGURE 3:**
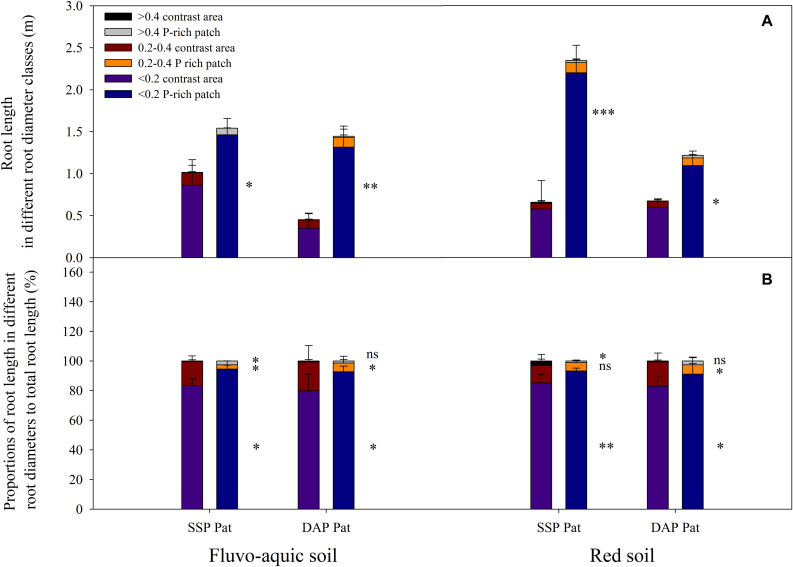
Root length in different root diameter classes **(A)** and proportions of root length in different root diameters to total root length **(B)** in fluvo-aquic soil and red soil with localized P supply. Asterisks indicate significant differences between P-rich patches and contrast areas in the same rhizobox (significance level: ^∗^*P* < 0.05; ^∗∗^*P* < 0.01; ^∗∗∗^*P* < 0.001; ns, no significant). Each column refers to the mean values of four replicates (+SD).

P application significantly improved maize growth (Figures 1A,B). In fluvo-aquic soil, the shoot biomass was 536 and 418% higher in SSP Hom and DAP Hom than in the control, respectively. There was no significant difference in plant biomass among SSP Pat, DAP Pat and the control. The total root biomass ranged from 0.12 to 0.55 g pot^–1^, which was significantly higher in SSP Hom and DAP Hom, with increases of 330 and 260%, respectively, than in the control. In red soil, the highest shoot biomass was observed in DAP Hom, which was 1.5 times that of SSP Hom and 2.6 times that of the control. In contrast, the total root biomass was the highest in SSP Hom (0.44 g pot^–1^), followed by DAP Hom and the other three treatments. No significant difference in either shoot or total root biomass among the control, SSP Pat, and DAP Pat was observed. Root mass ratio was significantly higher in control, SSP Pat, and DAP Pat compared with SSP Hom and DAP Hom and no significant difference in root tissue density among all treatments in both two types of soil (Supplementary Figure 2). The shoot P concentration in SSP Hom and DAP Hom was higher by 104 and 124% in fluvo-aquic soil and by 52 and 28% in red soil than in the control (Figures 1C,D). In fluvo-aquic soil, root P concentration was greater by 44% in SSP Hom and 64% in DAP Hom than in the control. In red soil, the root P concentration in the above two P Hom treatments was also 78 and 69% greater than that in the control, respectively. There was no significant difference in shoot and root P concentrations among the other remaining treatments. Significant root proliferation was observed in P-rich patches regardless of fertilizer type (Figure 2A). The root length was 54% higher in SSP patches and 220% higher in DAP patches than in contrast areas in the same rhizoboxes in fluvo-aquic soil and 258 and 75% higher in these patches in red soil, respectively. The specific root length in fluvo-aquic soil was 24% higher in SSP patches and 43% higher in DAP patches than in contrasting areas. However, no significant difference was observed between DAP patches and contrasting areas in red soil, and the specific root length was greater by 38% in SSP-rich patches than in contrasting areas (Figure 2B). Localized application of SSP and DAP significantly stimulated the proliferation of fine (small diameter) roots. The root length with a diameter < 0.2 mm in P-rich patches was significantly different from that in contrasting areas (Figure 3). It in SSP patches was 1.5 times higher in fluvo-aquic soil and 3.2 times higher in red soil than in contrasting areas and was 3.6 times and 1.8 times higher in DAP patches, respectively. The proportion of roots with a diameter < 0.2 mm in fluvo-aquic soil was 9.3% greater in the SSP patch and 13.7% greater in the DAP patch than in the corresponding contrasting areas. This proportion was approximately 8% greater in SSP and DAP patches than in contrasting areas in red soil.

The P concentration and content of roots in P-rich patches were significantly greater than those in contrasting areas (Figures 2C,D). In fluvo-aquic soil, the root P concentration in the SSP and DAP patches was 1.4 times and 1.7 times that in the contrasting areas, respectively. This concentrations in patches were 1.4 times and 2.0 times, respectively, than of contrasting areas in red soil. The root P content in SSP patches was greater than that in contrasting areas despite the soil type, and was 2.1 times that in fluvo-aquic soil and 2.7 times that in red soil. In the DAP patches, the P content was 2.6 times that in the contrasting area in the fluvo-aquic soil, but no significant increase was observed in the red soil.

**FIGURE 4:**
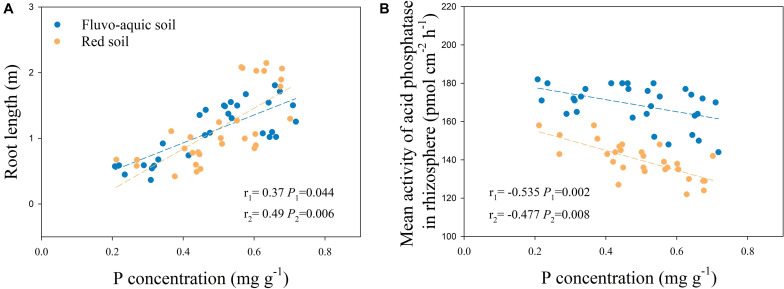
Correlation between root length and root P concentration in P-rich patches and contrast areas **(A)**, correlation between mean activity of acid phosphatase in rhizosphere and P concentration in P-rich patches and contrast areas **(B)**. r_1_: Correlation coefficient in fluvo-aquic soil, r_2_: Correlation coefficient in red soil.

In fluvo-aquic soil and red soil, root length in P-rich patches had a significant positive correlation with root P concentration, and the correlation coefficients were 0.37 (*P* = 0.044) and 0.49 (*P* = 0.006), respectively (Figure 4A).

### Diurnal Rhythm of APase Activity Across the Whole Soil Profile in Response to P Application

**FIGURE 5:**
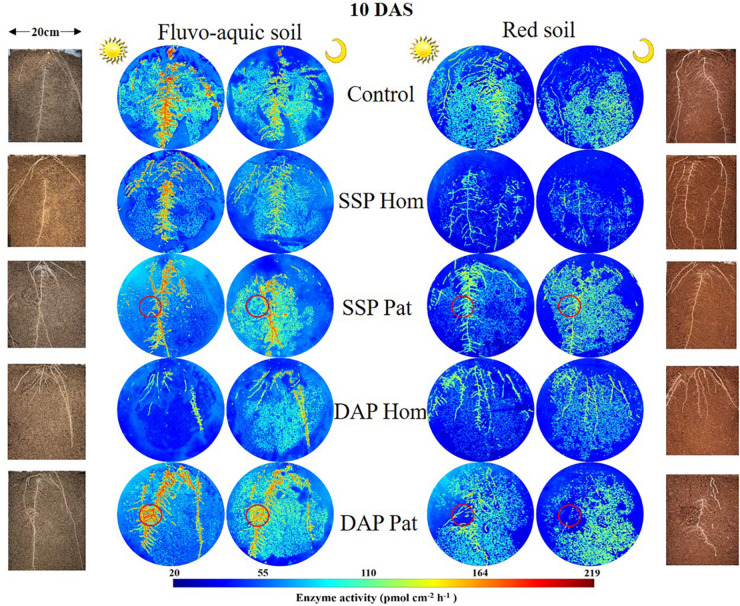
Distribution of acid phosphatase (middle) and maize roots grown in rhizoboxes (left and right), in fluvo-aquic soil and red soil, on 10 DAS. The first and third columns of zymogram were taken on noon, and the second and fourth columns of zymogram were taken at midnight. Lines from top to bottom represent maize grown in soil with no phosphorus addition, superphosphate homogenized application in the whole soil, superphosphate localized application in the P-rich patches (red circle), diammonium phosphate homogenized application in the whole soil, diammonium phosphate localized application in the P-rich patches (red circle), respectively. The color map is proportional to acid phosphatase activity (pmol cm^–2^ h^–1^).

**FIGURE 6:**
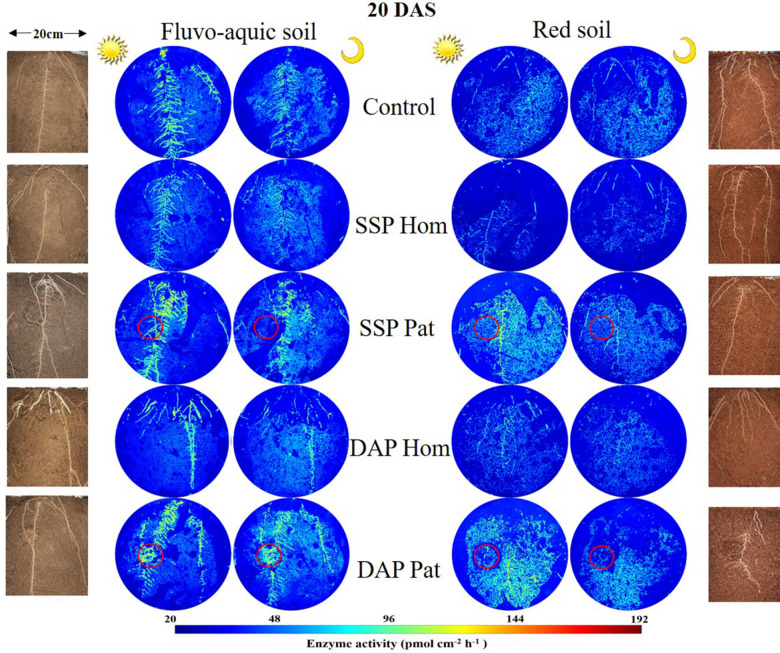
Distribution of acid phosphatase (middle) and maize roots grown in rhizoboxes (left and right), in fluvo-aquic soil and red soil, on 20 DAS. The first and third columns of zymogram were taken on noon, and the second and fourth columns of zymogram were taken at midnight. Lines from top to bottom represent maize grown in soil with no phosphorus addition, superphosphate homogenized application in the whole soil, superphosphate localized application in the P-rich patches (red circle), diammonium phosphate homogenized application in the whole soil, diammonium phosphate localized application in the P-rich patches, respectively (red circle). The color map is proportional to acid phosphatase activity (pmol cm^–2^ h^–1^).

**FIGURE 7:**
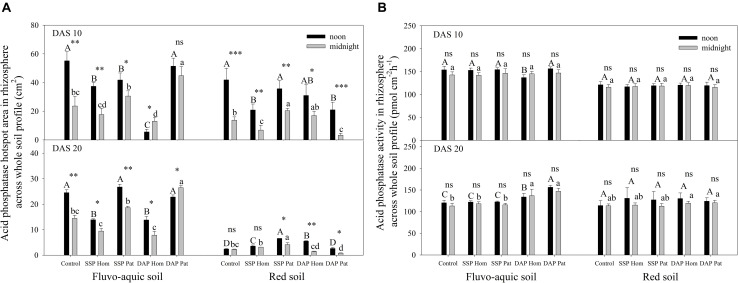
The hotspot area **(A)** and mean activity **(B)** of acid phosphatase in rhizosphere across whole soil profile on 10 DAS and 20 DAS in fluvo-aquic soil and red soil. Capital letters indicate significant differences between fertilization treatments on noon, lower-case letters indicate significant differences between fertilization treatments at midnight, and asterisks indicate significant differences between noon and midnight in the same rhizobox (significance level: ^∗^*P* < 0.05; ^∗∗^*P* < 0.01; ^∗∗∗^*P* < 0.001; ns, no significant). Each column refers to the mean values of four replicates (+SD).

**FIGURE 8:**
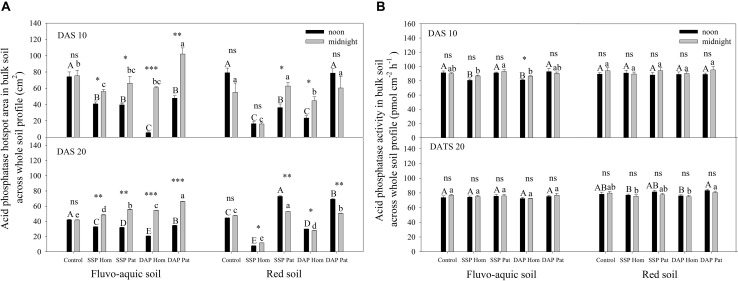
The hotspot area **(A)** and mean activity **(B)** of acid phosphatase in bulk soil across whole soil profile on 10 DAS and 20 DAS in fluvo-aquic soil and red soil. Capital letters indicate significant differences between fertilization treatments on noon, lower-case letters indicate significant differences between fertilization treatments at midnight, and asterisks indicate significant differences between noon and midnight in the same rhizobox (significance level: ^∗^*P* < 0.05; ^∗∗^*P* < 0.01; ^∗∗∗^*P* < 0.001; ns, no significant). Each column refers to the mean values of four replicates (+SD).

The spatiotemporal pattern of APase activity along and across the roots in different treatments on 10 and 20 days after sowing (DAS) was demonstrated by zymograms and is shown in Figures 5, 6. A larger hotspot area across the whole soil profile was observed in the control and P Pat treatments, which was 1.2–5 times as large as that under the P Hom treatments in both soil types (Figures 7A, 8A). In fluvo-aquic soil, APase mean activity in DAP Hom at noon was significantly lower than in other treatments in the rhizosphere at 10 DAS, and the lowest activity in bulk soil at both noon and midnight was observed in the P Hom treatments. In contrast, there was no significant difference in the mean activity of APase among all treatments in red soil. At 20 DAS, the mean APase activity in DAP Pat was greater than that in the other treatments in the rhizosphere of fluvo-aquic soil, and the activity in the P Hom treatments was significantly lower than that in the remaining treatments in red soil (Figures 7B, 8B). In fluvo-aquic soil, the hotspot area and mean activity of APase in rhizosphere and bulk soil were both greater than in red soil, irrespective of the growth period (Figures 7, 8). The hotspot areas of rhizosphere in fluvo-aquic soil were 1.3–13 times on 10 DAS and 4–29 times on 20 DAS as large as those in red soil. The APase hotspot area in rhizosphere and bulk soil showed an obvious opposite diurnal rhythm across the whole soil profile. The hot spot area in the rhizosphere was 10–140% higher at noon than at midnight (Figures 5, 6, 7A) but was 18–200% higher at midnight than at noon in bulk soil (Figures 5, 6, 8A). There was no significant difference in mean activity with the alternation of noon and midnight (Figures 7B, 8B). At 20 DAS, the hotspot area was more than 50% smaller, and the mean activity decreased by approximately 25 pmol cm^–2^ h^–1^ compared with that at 10 DAS in both types of soil (Figures 7, 8). This similar pattern was observed both in rhizosphere and bulk soil.

### Distribution of APase Activity Outward From the Root Axis in P-Rich Patches

**FIGURE 9:**
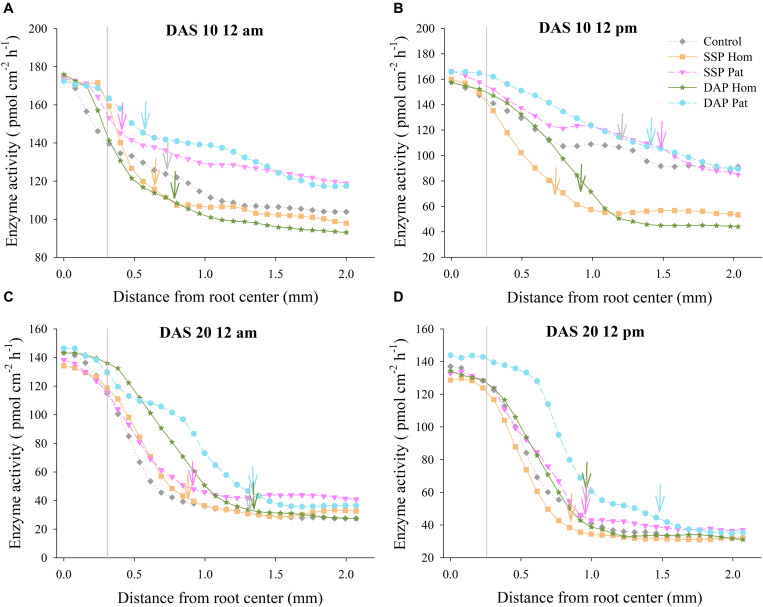
Acid phosphatase activity as a function of distance from root axis to the surrounding soil in P-rich patches. **(A–D)** represents different sampling time in fluvo-aquic soil, respectively. Vertical gray lines refers to average root radius, and vertical arrows represent rhizophere extension for enzyme activity. Each line calculated from four replicates, error bars are omitted to improve visualization.

**FIGURE 10:**
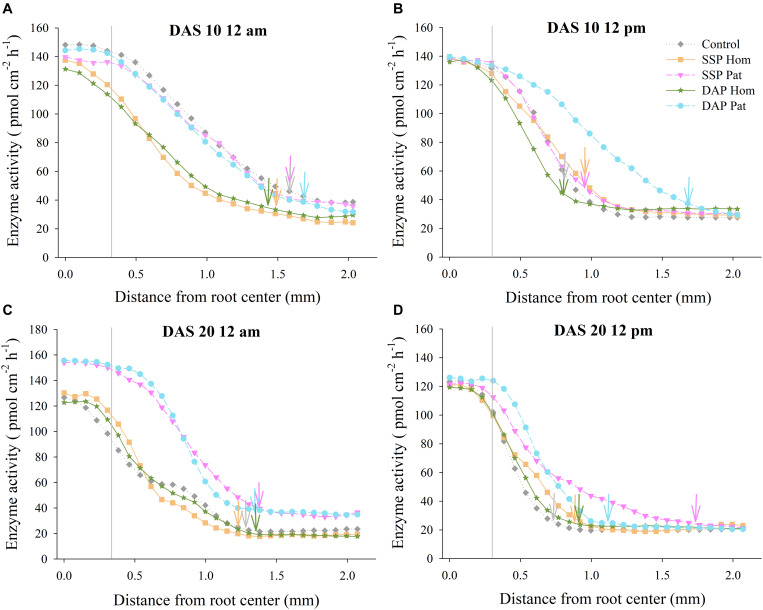
Acid phosphatase activity as a function of distance from root axis to the surrounding soil in P-rich patches. **(A–D)** represents different sampling time in red soil, respectively. Vertical gray lines refers to average root radius, and vertical arrows represent rhizophere extension for enzyme activity. Each line calculated from four replicates, error bars are omitted to improve visualization.

The distribution of APase across the roots is shown in Figures 5, 6. The activity of APase in adhered soils of roots was highest (Figures 9, 10). APase activity in P-rich patches declined slightly during a 0–0.3 mm distance from the root axis, but then, with increasing distance, enzyme activity decreased steeply to 25–90 pmol cm^–2^ h^–1^ (0.3–1.0 mm) until it became relatively stable and close to that of bulk soil (2 mm). In response to P deficiency, APase activity in the rhizosphere of individual roots in P-rich patches of the P Pat treatments was higher than that under the homogenized P supply treatments, especially at a 0.3–1.0 mm distance from the root axis (Figures 9, 10). Higher APase activity and broader rhizosphere extension were observed in the P Pat treatments than in the control and P Hom treatments. APase activity in the rhizosphere of P-rich patches followed a trend of DAP Pat ≥ SSP Pat > Control > SSP Hom = DAP Hom, regardless of sampling time and soil type. The localized application of DAP significantly enlarged the rhizosphere area in comparison with DAP Hom, and the extension ranged up to 0.91 mm from the root axis (Figure 10B); the maximum extension in SSP Pat was 0.84 mm (Figure 10D). Higher APase activity of individual roots was observed in fluvo-aquic soil than in red soil. In fluvo-aquic soil, the highest APase activity was close to 180 pmol cm^–2^ h^–1^ and 160 pmol cm^–2^ h^–1^ at noon and at midnight at 10 DAS, respectively (Figures 9A,B). In contrast, the highest activity in red soil was 140 pmol cm^–2^ h^–1^ (Figures 10A,B). The highest APase activity in the rhizosphere of individual roots also showed a diurnal pattern and was affected by the growth period; the highest activity was higher at noon than at midnight, and at 20 DAS, the highest APase activity decreased by approximately 20 pmol cm^–2^ h^–1^ (Figures 9, 10).

### APase Activity in P-Rich Patches

**FIGURE 11:**
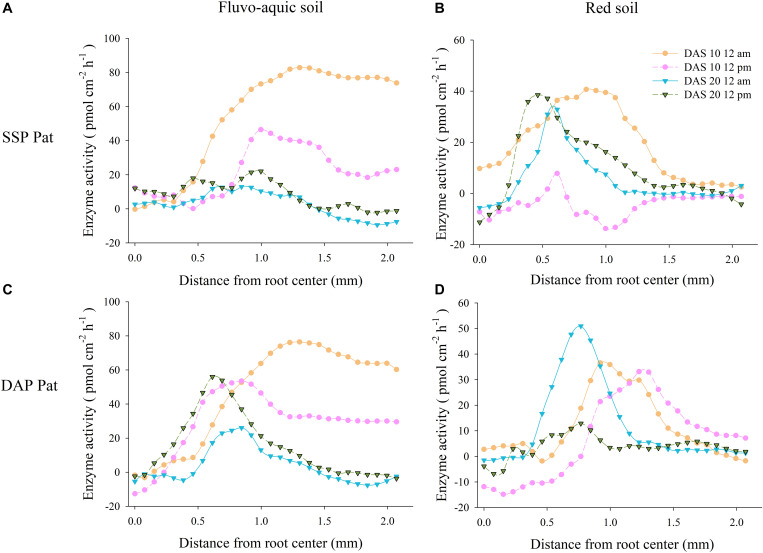
The difference between acid phosphatase activity in rhizosphere of P-rich patches and contrast areas with localized P supply. **(A,B)** Represents results of SSP Pat in fluvo-aquic soil and red soil, **(C,D)** represents results of DAP Pat in fluvo-aquic soil and red soil. Each line calculated from four replicates, error bars are omitted to improve visualization.

The APase activity in the rhizosphere was higher in P-rich patches than in contrasting areas regardless of P fertilizer application (Figure 11). The difference in APase activity between P-rich patches and contrasting areas was not significant near the root axis in all the treatments. However, the difference became larger with increasing distance from the root axis and finally reached a peak. The difference reached the peak in SSP Pat was 82.6 pmol cm^–2^ h^–1^ on 10 DAS at noon, 46.5 pmol cm^–2^ h^–1^ on 10 DAS at midnight, with 12.6 pmol cm^–2^ h^–1^ and 22.1 pmol cm^–2^ h^–1^ on 20 DAS at noon and midnight, respectively, in fluvo-aquic soil. The peaks in SSP Pat at 10 DAS at noon and midnight and 20 DAS at noon were approximately 40 pmol cm^–2^ h^–1^ at a 0.5–1 mm distance from the root axis in red soil (Figure 11B). In DAP Pat, the maximum difference was observed at a distance of 1.3 mm from the root axis at 10 DAS at noon (highest activity was 79 pmol cm^–2^ h^–1^) in fluvo-aquic soil. The difference at 20 DAS at midnight reached the highest point (25 pmol cm^–2^ h^–1^) at 0.6 mm from the root axis (Figure 11C). In red soil, the peak at 20 DAS was 0.2–0.45 mm nearer to the root axis than that at 10 DAS, and the maximum difference was 52 pmol cm^–2^ h^–1^ at 20 DAS at noon (Figure 11D).

There was a negative correlation between the mean activity of rhizosphere APase and root P concentration, and the correlation coefficients were −0.535 (*P* = 0.002) in fluvo-aquic soil and −0.477 (*P* = 0.008) in red soil (Figure 4B).

## Discussion

### Root Growth in P-Rich Patches

Under low P stress, plants allocate more carbohydrates to the root system, resulting in an increase in root mass ratio (Supplementary Figure 2; Hermans et al., 2006; Lynch, 2011). Localized nutrient (such as N and P) supplies significantly stimulate root proliferation and affect root configuration in these nutrient patches (Drew, 1975; Ma et al., 2007; Rabbi et al., 2017; Wang et al., 2018). We found that fine root proliferation contributed dominantly to an increase in root length in P-rich patches (Figure 3) and resulted in a higher specific root length in comparison with that in the contrasting area of the same rhizobox (Figure 2B). Since enhanced root branching and fine root proliferation not only expand the absorption surface of roots but can also facilitate P access in places where coarse roots cannot reach (Hutchings and de Kroon, 1994; Jing et al., 2010), the P concentration and content of roots in P-rich patches were higher than those in the contrasting regions (Figure 2).

### Distribution of APase Activity in Soil

Because APase activity is systemic regulated by shoot P status (Kummerova, 1986; Olander and Vitousek, 2000; Yadav and Tarafdar, 2001), it was higher in soils with a low P supply, such as those of the control, SSP Pat, and DAP Pat treatments, than that in soils with a relatively high P supply (Figures 5, 6; Tarafdar and Claassen, 1988; Miller et al., 2001). The strong adsorption capacity of minerals causes an underestimation of APase activity in acidic soils by zymograms and histograms (Poll et al., 2006). This was one of the possible reasons why APase activity was lower in red soil than in fluvo-aquic soil in this study. Moreover, a high shoot P concentration was also proposed to depress APase secretion from roots in red soil (Figure 1C). The growth stages of crops are another factor influencing APase activity in the rhizosphere. In both Yadav and Tarafdar’s (2001) and our study (Figure 7), rhizosphere APase activity declined with maize growth. We found that the mean activity only marginally decreased, but hotspot areas were strongly affected by the growth stage (Figures 7A,B). This result indicated that the decline in enzyme activity was mainly due to the reduction in secretion of the enzyme, not the other factors, such as pH mentioned by Rudolph-Mohr et al. (2017) and status of plant P nutrition mentioned by Schimel and Weintraub (2003) in this study.

The APase activity in both rhizosphere and bulk soils changed spatially and temporally during crop growth in this study (Figures 5, 6). The APase activity decreased with increasing distance from the root axis. Two reasons contributed to this distribution pattern. First, the APase secreted by roots tends to accumulate near roots because of its low diffusion rate (Asmar et al., 1994). Second, soil microorganisms, as another important source of APase, are also concentrated in the rhizosphere, where organic carbon is always richer than in bulk soil (Kuzyakov and Domanski, 2000).

**FIGURE 12:**
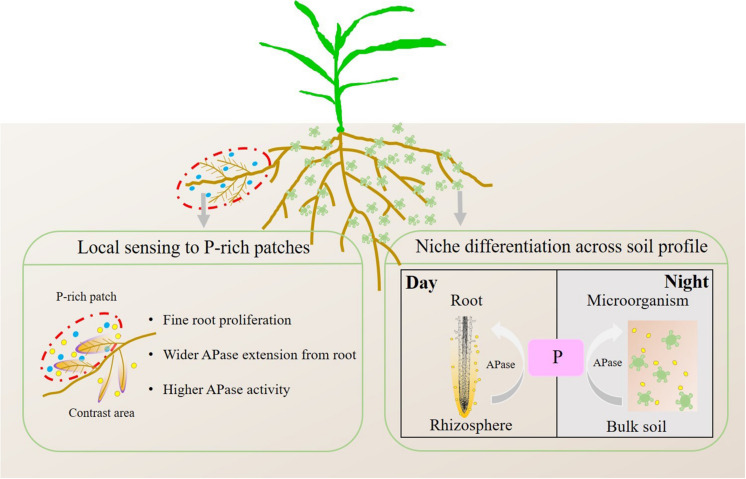
Conceptual model of root growth and APase activity in soil sensing to P-rich patches. The blue circles represent P fertilizer, the yellow circles represent APase secreted by roots and microorganisms, and the green irregular shapes represent microorganisms.

In contrast to the mean activity of APase in soils, the APase activity of individual roots increased with localized P application (Figures 9, 10), which supports the first hypothesis (H1). Localized P treatments showed a greater rhizosphere extension than the control and homogenized P supply treatments, indicating that, as the hotspot area, the extension of the APase rhizosphere was also inhibited by homogenous P application. The extension of APase played a vital role in the mobilization of soil P since each 1 mm increase in the rhizosphere area resulted in a more than 3-fold increase in the soil volume (Razavi et al., 2017; Wei et al., 2018). We found that the APase activity in the rhizosphere of individual roots was higher in DAP patches and SSP patches than in contrasting areas. This result is contrary to what Funakoshi proposed in 2018, the author found APase activities decreased in densely branched lateral roots (DBLRs), but were significantly higher in non-DBLR in the same plants when mineral P fertilizer local applied at a rate of 10 mg kg^–1^ soil, however such significant difference became smaller as the concentration of P increased to 100 mg kg^–1^ soil. They associated to P deficiency in DBLR cells was alleviated by P acquisition, in contrast, non-DBLR roots likely to recycle P (Funakoshi et al., 2018). Shen et al. (2005) cultured white lupine in hydroponic system with two compartments, APase activities were higher in the root half in −P−P than those in +P+P or −P+P, but no significant difference in the exudation rates of APase between two root halves in −P + P despite the increased exudation rate of APase at 0 mM P compared with 250 mM P (Shen et al., 2005). This phenomenon can be attributed to an inhibiting effect of higher P in plants on the exudation of APase. However, our result indicated that APase activity in the rhizosphere was directly regulated by the localized P supply, not indirectly through changes in the root P concentration causing feedback because they showed a negative correlation (Figure 4B) in these treatments, which means under the regulation of local signal, more APase is released in the patch area compared to the contrast area. High soil labile P can inhibit bacterial phosphatase activity in soil (Sinsabaugh et al., 2008; Fraser et al., 2017); thus, the increase in APase activity in P-rich patches should not be due to the secretion of soil microorganisms. Consistent with Spiers’ conclusion, in the case of overall P deficiency, P-rich patch may further stimulate the secretion of APase in patch area to encourage plants to obtain as much of the limited soil-derived P as possible, and therefore offset the initial inhibition caused by the inorganic fertilizer. But this phenomenon may be affected by the cultivate system, crop type, P application rate and supply method. Another possible reason is that the organic P and inorganic P resources in nature often coexist, plants may not be able to clearly distinguish the source of P and can only through the growth needs of plant to determine whether to secrete more APase. Therefore, we concluded that even a localized P supply can enhance the mobilization of soil organic P by roots. Based on the results in this study, we propose a conceptual model of root growth and APase activity in soil in response to P-rich patches (Figure 12).

### Temporal Variation of APase Activity in Soil

In line with the second hypothesis (H2), APase activity had a diurnal pattern (Figures 5, 6). However, the APase activity in the rhizosphere and bulk soil showed opposite diurnal rhythms (Figures 7, 8). Photosynthesis takes place during the day and produces large amounts of carbohydrates that are transported to roots to support physiological and biochemical processes (Kuzyakov and Cheng, 2001), including APase secretion. This was why APase activity in the rhizosphere was higher at noon than at midnight. Mishra and Panda (1970) also found that the significantly increased phosphatase activity of leaves at noon is related to the diurnal variation of stomatal opening and transpiration in leaves (Mishra and Panda, 1970). Many studies have shown that the diurnal cycling of rhizosphere bacterial community structures and functions can be adjusted by the plant circadian clock (Hubbard et al., 2017; Staley et al., 2017). In this study, we found that APase activity in bulk soil also showed a diurnal rhythm, being high at midnight and low at noon. This may be associated with the diurnal dynamics influencing plant-microbe carbon metabolism and allocation. Maximal production of sugars, only a fraction of which are released to soil (Lu et al., 2005; Bais et al., 2006), occurs during the light period. Conversely, genes that regulate the transport of sugars reach their maximum expression levels at dusk, and more carbohydrates are released into the soil to maintain carbon homeostasis in the rhizosphere (Harmer et al., 2000); therefore, microbial activity is enhanced at night, indicating that roots and microorganisms can alleviate competition not only through spatial separation but also through the circadian rhythm. Previous studies also showed that the microbial community structure and abundance may vary depending on day vs. night soil water or nutrient availability, since the concentration of some exudates (such as amino acids, organic acids, flavonols and so on) (Watt and Evans, 1999; Badri and Vivanco, 2009) and the nutrient flow affected by transpiration (Matimati et al., 2014) varied over the course of the day. APase activity in the rhizosphere during the day was dominantly driven by plants, and that in bulk soil at midnight was mainly driven by soil microorganisms. We believe this niche differentiation of soil organic P mobilization between plants and soil microorganisms on spatial and temporal scales (Figure 12) can facilitate P utilization of the whole plant-microorganism system by alleviating intercompetition.

Since the APase secreted by roots is not easily distinguished from by microorganisms in this study, further study may can carry in sterile soil or use soil zymography to visualize the temporal and spatial distribution of alkaline phosphatase, which secreted only by microorganisms (Juma and Tabatabai, 1988; Nannipieri et al., 2011).

## Conclusion

We used soil zymography to visualize *in situ* APase activity in rhizosphere and bulk soil of maize with different P application strategies. We found that (1) APase secretion of roots was plastic, it can be induced by P-rich patches, indicating that under P stress if even mineral P fertilizers are localized, mobilization of soil organic P by roots can also be enhanced; (2) The activity of APase had a spatiotemporal rhythm, APase activity in the rhizosphere was dominantly driven by plants, and that in bulk soil was mainly driven by soil microorganisms, they showed the opposite diurnal rhythm. Additionally, we believe the opposite diurnal rhythms will result in not only spatial but temporal niche differentiation for mobilization of soil organic P, which alleviated competition between plants and microorganisms, but this hypothesis needs to be proved by further experiments in the future.

## Data Availability Statement

All datasets generated for this study are included in the article/Supplementary Material, further inquiries can be directed to the corresponding author/s.

## Author Contributions

XM, JZ, and HL: conceptualization. XM: methodology, formal analysis and investigation, and writing—original draft preparation. XM, JZ, JS, and HL: writing—review and editing. HL: funding acquisition. All authors contributed to the article and approved the submitted version.

## Conflict of Interest

The authors declare that the research was conducted in the absence of any commercial or financial relationships that could be construed as a potential conflict of interest.

## Supplementary Material

Supplementary Figure 1 Schematic diagram of the experimental rhizobox and the location of the P-rich patch (the brown cycle).

Supplementary Figure 2 Root mass ratio **(A)** and root tissue density **(B)** of maize on 30 DAS in fluvo-aquic soil and red soil. Capital letters indicate significant differences between different soil types, and lower-case letters indicate significant differences between fertilization treatments. Each column refers to the mean value of four replicates (+SD).

## References

[B71] Anderson G. (1980). “Assessing organic phosphorus in soils,” in *Role of Phosphorus in Agriculture*, eds Khasawneh F. E. Sample E. C. Kamprath E. J. (Madison, WI: American Society for Agronomy), 411–431. 10.2134/1980.roleofphosphorus.c16

[B1] Asmar F. Eiland F. Nielsen N. E. (1994). Effect of extracellular-enzyme activities on solubilization rate of soil organic nitrogen. *Biol. Fertil. Soils* 17 32–38. 10.1007/bf00418669

[B2] Azeem K. Khan A. Naz F. Ilyas M. Azeem I. Anwar F. (2018). The impact of different P fertilizer sources on growth, yield and yield component of maize varieties. *Agric. Res. Technol.* 13 55–58.

[B3] Badri D. V. Vivanco J. M. (2009). Regulation and function of root exudates. *Plant Cell Environ.* 32 666–681. 10.1111/j.1365-3040.2009.01926.x 19143988

[B4] Bais H. P. Weir T. L. Perry L. G. Gilroy S. Vivanco J. M. (2006). The role of root exudates in rhizosphere interactions with plants and other organisms. *Annu. Rev. Plant Biol.* 57 233–266. 10.1146/annurev.arplant.57.032905.105159 16669762

[B5] Blossfeld S. Schreiber C. M. Liebsch G. Kuhn A. J. Hinsinger P. (2013). Quantitative imaging of rhizosphere pH and CO_2_ dynamics with planar optodes. *Ann. Bot.* 112 267–276. 10.1093/aob/mct047 23532048 PMC3698388

[B6] Cordell D. Drangert J. O. White S. (2009). The story of phosphorus: global food security and food for thought. *Glob. Environ. Change* 19 292–305. 10.1016/j.gloenvcha.2008.10.009

[B7] Corrales I. Amenos M. Poschenrieder C. Barcelo J. (2007). Phosphorus efficiency and root exudates in two contrasting tropical maize varieties. *J. Plant Nutr.* 30 887–900. 10.1080/15226510701375085

[B8] Dong S. Brooks D. Jones M. D. Grayston S. J. (2007). A method for linking in situ activities of hydrolytic enzymes to associated organisms in forest soils. *Soil Biol. Biochem.* 39 2414–2419. 10.1016/j.soilbio.2007.03.030

[B9] Drew M. C. (1975). Comparison of the effects of a localized supply of phosphate, nitrate, ammonium and potassium on the growth of the seminal root system, and the shoot, in barley. *New Phytol.* 75 479–490. 10.1111/j.1469-8137.1975.tb01409.x

[B10] Fraser T. D. Lynch D. H. Gaiero J. Khosla K. Dunfield K. E. (2017). Quantification of bacterial non-specific acid (phoC) and alkaline (phoD) phosphatase genes in bulk and rhizosphere soil from organically managed soybean fields. *Appl. Soil Ecol.* 111 48–56. 10.1016/j.apsoil.2016.11.013

[B11] Funakoshi Y. Daimon H. Matsumura A. (2018). Morphological and physiological studies on densely branched lateral roots triggered by localized phosphate in *Sesbania cannabina*. *J. Plant Nutr. Soil Sci.* 181 336–344. 10.1002/jpln.201700228

[B12] Ge X. Cao Y. Zhou B. Wang X. Li M. H. J. A. S. E. (2019). Biochar addition increases subsurface soil microbial biomass but has limited effects on soil CO_2_ emissions in subtropical moso bamboo plantations. *Appl. Soil Ecol.* 142 155–165. 10.1016/j.apsoil.2019.04.021

[B13] George T. S. Gregory P. J. Robinson J. S. Buresh R. J. (2002). Changes in phosphorus concentrations and pH in the rhizosphere of some agroforestry and crop species. *Plant Soil* 246 65–73. 10.1023/a:1021523515707

[B14] Giles C. D. Dupuy L. Boitt G. Brown L. K. Condron L. M. Darch T. (2018). Root development impacts on the distribution of phosphatase activity: improvements in quantification using soil zymography. *Soil Biol. Biochem.* 116 158–166. 10.1016/j.soilbio.2017.08.011

[B15] Guber A. Kraychenko A. Razavi B. S. Uteau D. Peth S. Blagodatskaya E. (2018). Quantitative soil zymography: mechanisms, processes of substrate and enzyme diffusion in porous media. *Soil Biol. Biochem.* 127 156–167. 10.1016/j.soilbio.2018.09.030

[B16] Harmer S. L. Hogenesch J. B. Straume M. Chang H. S. Han B. Zhu T. (2000). Orchestrated transcription of key pathways in *Arabidopsis* by the circadian clock. *Science* 290 2110–2113. 10.1126/science.290.5499.2110 11118138

[B17] Hermans C. Hammond J. P. White P. J. Verbruggen N. (2006). How do plants respond to nutrient shortage by biomass allocation? *Trends Plant Sci.* 11 610–617. 10.1016/j.tplants.2006.10.007 17092760

[B18] Hinsinger P. (2001). Bioavailability of soil inorganic P in the rhizosphere as affected by root-induced chemical changes: a review. *Plant Soil* 237, 173–195. 10.1023/A:1013351617532

[B19] Hoang D. T. T. Razavi B. S. Kuzyakov Y. Blagodatskaya E. (2016). Earthworm burrows: kinetics and spatial distribution of enzymes of C-, N- and P- cycles. *Soil Biol. Biochem.* 99 94–103. 10.1016/j.soilbio.2016.04.021

[B20] Hubbard C. J. Brock M. T. Diepen L. T. V. Maignien L. Weinig C. (2017). The plant circadian clock influences rhizosphere community structure and function. *ISME J.* 12 400–410. 10.1038/ismej.2017.172 29053146 PMC5776454

[B21] Hutchings M. J. de Kroon H. (1994). “Foraging in plants: the role of morphological plasticity in resource acquisition,” in *Advances in Ecological Research*, eds Begon M. Fitter A. H. (Cambridge, MA: Academic Press), 159–238. 10.1016/s0065-2504(08)60215-9

[B22] Jakobsen I. Leggett M. E. Richardson A. E. (2005). “Rhizospheremicroorganisms and plant phosphorus uptake,” in *Phosphorus, Agriculture and the Environment*, eds Sims J. T. Sharpley A. N. (Madison, WI: American Society for Agronomy), 437–494. 10.2134/agronmonogr46.c14

[B23] Jing J. Rui Y. Zhang F. Rengel Z. Shen J. (2010). Localized application of phosphorus and ammonium improves growth of maize seedlings by stimulating root proliferation and rhizosphere acidification. *Field Crops Res.* 119 355–364. 10.1016/j.fcr.2010.08.005

[B24] Juma N. G. Tabatabai M. A. (1988). Hydrolysis of organic-phosphates by corn and soybean roots. *Plant Soil* 107 31–38. 10.1007/bf02371541

[B25] Khan S. U. (1970). Enzymatic activity in a gray wooded soil as influenced by cropping systems and fertilizers. *Soil Biol. Biochem.* 2, 137–139. 10.1016/0038-0717(70)90016-7

[B26] Koch O. Tscherko D. Kandeler E. (2007). Temperature sensitivity of microbial respiration, nitrogen mineralization, and potential soil enzyme activities in organic alpine soils. *Glob. Biogeochem. Cycles* 21 223–228. 10.1029/2007gb002983

[B27] Kummerova M. (1986). Localization of acid-phosphatase-activity in maize root under phosphorus deficiency. *Biol. Plant.* 28 270–274. 10.1007/bf02902291

[B28] Kuzyakov Y. Blagodatskaya E. (2015). Microbial hotspots and hot moments in soil: concept & review. *Soil Biol. Biochem.* 83 184–199. 10.1016/j.soilbio.2015.01.025

[B29] Kuzyakov Y. Cheng W. (2001). Photosynthesis controls of rhizosphere respiration and organic matter decomposition. *Soil Biol. Biochem.* 33 1915–1925. 10.1016/s0038-0717(01)00117-1

[B30] Kuzyakov Y. Domanski G. (2000). Carbon input by plants into the soil. review. *J. Plant Nutr. Soil Sci.* 163 421–431. 10.1016/s0038-0717(01)00117-1

[B31] Liu S. B. Razavi B. S. Su X. Maharjan M. Zarebanadkouki M. Blagodatskaya E. (2017). Spatio-temporal patterns of enzyme activities after manure application reflect mechanisms of niche differentiation between plants and microorganisms. *Soil Biol. Biochem.* 112 100–109. 10.1016/j.soilbio.2017.05.006

[B32] Lu Y. Gehan J. P. Sharkey T. D. (2005). Daylength and circadian effects on starch degradation and maltose metabolism. *Plant Physiol.* 138 2280–2291. 10.1104/pp.105.061903 16055686 PMC1183414

[B33] Lynch J. P. (2011). Root phenes for enhanced soil exploration and phosphorus acquisition: tools for future crops. *Plant Physiol.* 156 1041–1049. 10.1104/pp.111.175414 21610180 PMC3135935

[B34] Ma Q. Rengel Z. Bowden B. (2007). Heterogeneous distribution of phosphorus and potassium in soil influences wheat growth and nutrient uptake. *Plant Soil* 291 301–309. 10.1007/s11104-007-9197-5

[B35] Ma X. Zarebanadkouki M. Kuzyakov Y. Blagodatskaya E. Pausch J. Razavi B. S. (2018). Spatial patterns of enzyme activities in the rhizosphere: effects of root hairs and root radius. *Soil Biol. Biochem.* 118 69–78. 10.1016/j.soilbio.2017.12.009

[B36] Marschner P. (2013). *Marschner’s Mineral Nutrition of Higher Plants*, 2nd Edn. London: Academic Press.

[B37] Matimati I. Verboom G. A. Cramer M. D. (2014). Do hydraulic redistribution and nocturnal transpiration facilitate nutrient acquisition in *Aspalathus linearis*? *Oecologia* 175 1129–1142. 10.1007/s00442-014-2987-6 24972698

[B38] Miller S. S. Liu J. Q. Allan D. L. Menzhuber C. J. Fedorova M. Vance C. P. (2001). Molecular control of acid phosphatase secretion into the rhizosphere of proteoid roots from phosphorus-stressed white lupin. *Plant Physiol.* 127 594–606. 10.1104/pp.127.2.594 11598233 PMC125094

[B39] Mishra D. Panda K. C. (1970). Acid phosphatases of rice leaves showing diurnal variation and its relation to stomatal opening. *Biochem. Physiol. Pflanzen* 161 532–536. 10.1016/S0015-3796(17)31088-0

[B40] Nannipieri P. Giagnoni L. Landi L. Renella G. (2011). *Role of Phosphatase Enzymes in Soil.* Berlin: Springer.

[B41] Neumann G. Martinoia E. A. Romheld V. (1999). Physiological adaptations to phosphorus deficiency during proteoid root development in white lupin. *Planta* 208 373–382. 10.1007/s004250050572

[B42] Olander L. P. Vitousek P. M. (2000). Regulation of soil phosphatase and chitinase activity by N and P availability. *Biogeochemistry* 49 175–190. 10.1023/a:1006316117817

[B43] Poll C. Ingwersen J. Stemmer M. Gerzabek M. H. Kandeler E. (2006). Mechanisms of solute transport affect small-scale abundance and function of soil microorganisms in the detritusphere. *Eur. J. Soil Sci.* 57 583–595. 10.1111/j.1365-2389.2006.00835.x

[B44] Rabbi S. M. F. Guppy C. N. Tighe M. K. Flavel R. J. Young I. M. (2017). Root architectural responses of wheat cultivars to localised phosphorus application are phenotypically similar. *J. Plant Nutr. Soil Sci.* 180 169–177. 10.1002/jpln.201600503

[B45] Razavi B. S. Hoang D. T. T. Blagodatskaya E. Kuzyakov Y. (2017). Mapping the footprint of nematodes in the rhizosphere: cluster root formation and spatial distribution of enzyme activities. *Soil Biol. Biochem.* 115 213–220. 10.1016/j.soilbio.2017.08.027

[B46] Razavi B. S. Zarebanadkouki M. Blagodatskaya E. Kuzyakov Y. (2016). Rhizosphere shape of lentil and maize: spatial distribution of enzyme activities. *Soil Biol. Biochem.* 96 229–237. 10.1016/j.soilbio.2016.02.020

[B47] Rudolph N. Voss S. Moradi A. B. Nagl S. Oswald S. E. (2013). Spatio-temporal mapping of local soil pH changes induced by roots of lupin and soft-rush. *Plant Soil* 369 669–680. 10.1007/s11104-013-1775-0

[B48] Rudolph-Mohr N. Tötzke C. Kardjilov N. Oswald S. E. (2017). Mapping water, oxygen, and pH dynamics in the rhizosphere of young maize roots. *J. Plant Nutr. Soil Sci.* 180 336–346. 10.1002/jpln.201600120

[B49] Schachtman D. P. Reid R. J. Ayling S. M. (1998). Phosphorus uptake by plants: from soil to cell. *Plant Physiol.* 116 447–453. 10.1104/pp.116.2.447 9490752 PMC1539172

[B50] Schimel J. P. Weintraub M. N. (2003). The implications of exoenzyme activity on microbial carbon and nitrogen limitation in soil: a theoretical model. *Soil Biol. Biochem.* 35 549–563. 10.1016/s0038-0717(03)00015-4

[B51] Shen J. Li H. Neumann G. Zhang F. (2005). Nutrient uptake, cluster root formation and exudation of protons and citrate in *Lupinus albus* as affected by localized supply of phosphorus in a split-root system. *Plant Sci.* 168 837–845. 10.1016/j.plantsci.2004.10.017

[B52] Sinsabaugh R. L. Lauber C. L. Weintraub M. N. Ahmed B. Allison S. D. Crenshaw C. (2008). Stoichiometry of soil enzyme activity at global scale. *Ecol. Lett.* 11 1252–1264. 10.1111/j.1461-0248.2008.01245.x 18823393

[B53] Spiers G. A. Mcgill W. B. (1979). Effects of phosphorus addition and energy supply on acid phosphatase production and activity in soils. *Soil Biol. Biochem.* 11, 3–8. 10.1016/0038-0717(79)90110-X

[B54] Staley C. Ferrieri A. P. Tfaily M. M. Cui Y. Chu R. K. Wang P. (2017). Diurnal cycling of rhizosphere bacterial communities is associated with shifts in carbon metabolism. *Microbiome* 5:65. 10.1186/s40168-017-0287-1 28646918 PMC5483260

[B55] Stryker R. B. Gilliam J. W. Jackson W. A. (1974). Nonuniform phosphorus distribution in the root zone of corn: growth and phosphorus uptake 1. *Soil Sci. Soc. Am. J.* 38 334–340. 10.2136/sssaj1974.03615995003800020034x

[B56] Tarafdar J. C. Claassen N. (1988). Organic phosphorus-compounds as a phosphorus source for higher-plants through the activity of phosphatases produced by plant-roots and microorganisms. *Biol. Fertil. Soils* 5 308–312.

[B57] Tiessen H. (2008). “Phosphorus in the global environment,” in *The Ecophysiology of Plant-Phosphorus Interactions*, eds White P. J. Hammond J. P. (Berlin: Springer), 1–7. 10.1007/978-1-4020-8435-5_1

[B58] Tomscha J. L. Trull M. C. Deikman J. Lynch J. P. Guiltinan M. J. (2004). Phosphatase under-producer mutants have altered phosphorus relations. *Plant Physiol.* 135 334–345. 10.1104/pp.103.036459 15122033 PMC429387

[B59] Vance C. P. Uhde-Stone C. Allan D. L. (2003). Phosphorus acquisition and use: critical adaptations by plants for securing a nonrenewable resource. *New Phytol.* 157 423–447. 10.1046/j.1469-8137.2003.00695.x 33873400

[B60] Walter A. Pich A. Scholz G. Marschner H. Romheld V. J. (1995). Effects of iron nutritional status and time of day on concentrations of phytosiderophores and nico-tianamine in different root and shoot zones of barley. *J. Plant Nutr.* 18 1577–1593. 10.1080/01904169509365005

[B61] Wang Y. Jensen L. S. Magid J. (2018). Low-P solution culture can be used for screening root growth vigor in soil for high nutrient uptake of spring wheat varieties. *Acta Agric. Scand. B Soil Plant Sci.* 68 130–138. 10.1080/09064710.2017.1367835

[B62] Watt M. Evans J. R. (1999). Linking development and determinacy with organic acid efflux from proteoid roots of white lupin grown with low phosphorus and ambient or elevated atmospheric CO_2_ concentration. *Plant Physiol.* 120 705–716. 10.1104/pp.120.3.705 10398705 PMC59308

[B63] Wei X. Ge T. Zhu Z. Hu Y. Liu S. Li Y. (2018). Expansion of rice enzymatic rhizosphere: temporal dynamics in response to phosphorus and cellulose application. *Plant Soil* 445 169–181. 10.1007/s11104-018-03902-0

[B64] Wei X. Razavi B. S. Hu Y. Xu X. Zhu Z. Liu Y. (2019). C/P stoichiometry of dying rice root defines the spatial distribution and dynamics of enzyme activities in root-detritusphere. *Biol. Fertil. Soils* 55 251–263. 10.1007/s00374-019-01345-y

[B65] Wen Z. Li H. Shen J. Rengel Z. (2017). Soil maize responds to low shoot P concentration by altering root morphology rather than increasing root exudation. *Plant Soil* 416 377–389. 10.1007/s11104-017-3214-0

[B66] Westerman R. L. (1990). “Soil testing and plant analysis,” in *Soil Science Society of America Book Series*, ed. Westerman R. L. (New York, NY: Wiley), 223–236.

[B67] Xu X. Liu X. Li Y. Ran Y. Liu Y. Zhang Q. (2017). High temperatures inhibited the growth of soil bacteria and archaea but not that of fungi and altered nitrous oxide production mechanisms from different nitrogen sources in an acidic soil. *Soil Biol. Biochem.* 107 168–179. 10.1016/j.soilbio.2017.01.003

[B68] Yadav R. S. Tarafdar J. C. (2001). Influence of organic and inorganic phosphorus supply on the maximum secretion of acid phosphatase by plants. *Biol. Fertil. Soils* 34 140–143. 10.1007/s003740100376

[B69] Zhang W. Tang X. Feng X. Wang E. Li H. Shen J. (2019). Management strategies to optimize soil phosphorus utilization and alleviate environmental risk in China. *J. Environ. Qual.* 48 1167–1175. 10.2134/jeq2019.02.0054 31589723

[B70] Zhang Y. Yu P. Peng Y.-F. Li X.-X. Chen F.-J. Li C. J. (2012). Fine root patterning and balanced inorganic phosphorus distribution in the soil indicate distinctive adaptation of maize plants to phosphorus deficiency. *Pedosphere* 22 870–877. 10.1016/s1002-0160(12)60073-3

