# Supplementary material for: Spatiotemporal Pattern of Acid Phosphatase Activity in Soils Cultivated With Maize Sensing to Phosphorus-Rich Patches
Source: Front Plant Sci. 2021 Apr 13;12:650436. doi: 10.3389/fpls.2021.650436 (PMC8076754; doi:10.3389/fpls.2021.650436)
Supplement: Supplementary Figure 1 — Schematic diagram of the experimental rhizobox and the location of the P-rich patch (the brown cycle). [file Data_Sheet_1.docx]

**Electronic supplementary material**

**Supplementary Figure. S1**
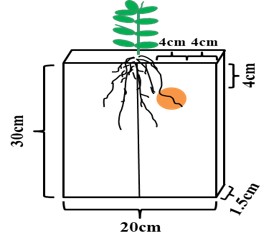


**Figure. S1** Schematic diagram of the experimental rhizobox and the location of the P-rich patch (the brown cycle)


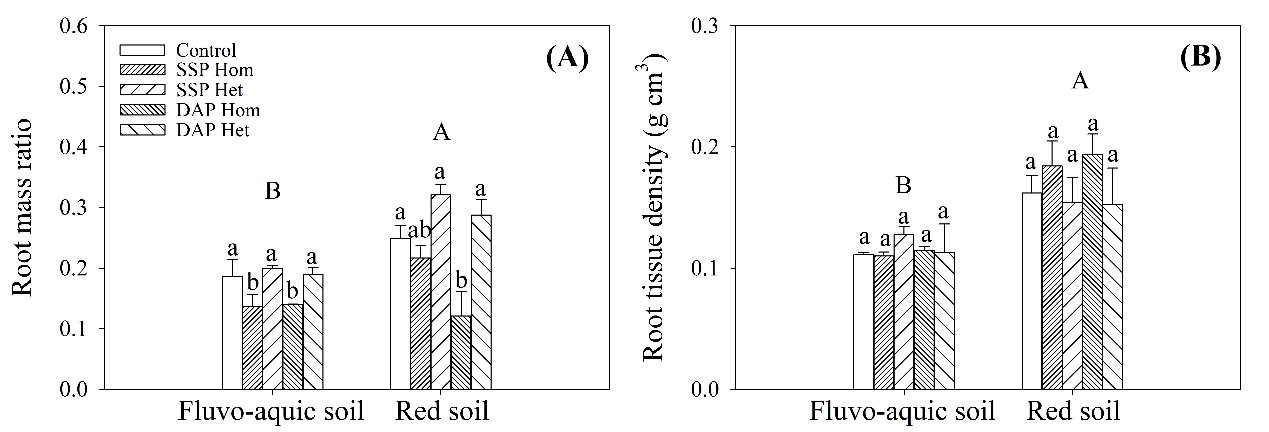
**Supplementary Figure. S2**

**Fig. S2** Root mass ratio (A) and root tissue density (B) of maize on 30 DAS in fluvo-aquic soil and red soil. Capital letters indicate significant differences between different soil types, and lower-case letters indicate significant differences between fertilization treatments. Each column refers to the mean value of four replicates (+SD)
